# Verification of a Method for Measuring Parkinson’s Disease Related Temporal Irregularity in Spiral Drawings

**DOI:** 10.3390/s17102341

**Published:** 2017-10-13

**Authors:** Somayeh Aghanavesi, Mevludin Memedi, Mark Dougherty, Dag Nyholm, Jerker Westin

**Affiliations:** 1 School of Technology and Business Studies, Computer Engineering, Dalarna University, 791 88 Falun, Sweden; mdo@du.se (M.D.); jwe@du.se (J.W.); 2 Informatics, Business School, Örebro University, 702 81 Örebro, Sweden; Mevludin.Memedi@oru.se; 3 Department of Neuroscience, Neurology, Uppsala University, 751 85 Uppsala, Sweden; dag.nyholm@neuro.uu.se

**Keywords:** Parkinson’s disease, smartphone, spiral tests, temporal irregularity, timing variability, motor assessment, approximate entropy, complexity

## Abstract

Parkinson’s disease (PD) is a progressive movement disorder caused by the death of dopamine-producing cells in the midbrain. There is a need for frequent symptom assessment, since the treatment needs to be individualized as the disease progresses. The aim of this paper was to verify and further investigate the clinimetric properties of an entropy-based method for measuring PD-related upper limb temporal irregularities during spiral drawing tasks. More specifically, properties of a temporal irregularity score (TIS) for patients at different stages of PD, and medication time points were investigated. Nineteen PD patients and 22 healthy controls performed repeated spiral drawing tasks on a smartphone. Patients performed the tests before a single levodopa dose and at specific time intervals after the dose was given. Three movement disorder specialists rated videos of the patients based on the unified PD rating scale (UPDRS) and the Dyskinesia scale. Differences in mean TIS between the groups of patients and healthy subjects were assessed. Test-retest reliability of the TIS was measured. The ability of TIS to detect changes from baseline (before medication) to later time points was investigated. Correlations between TIS and clinical rating scores were assessed. The mean TIS was significantly different between healthy subjects and patients in advanced groups (*p*-value = 0.02). Test-retest reliability of TIS was good with Intra-class Correlation Coefficient of 0.81. When assessing changes in relation to treatment, TIS contained some information to capture changes from Off to On and wearing off effects. However, the correlations between TIS and clinical scores (UPDRS and Dyskinesia) were weak. TIS was able to differentiate spiral drawings drawn by patients in an advanced stage from those drawn by healthy subjects, and TIS had good test-retest reliability. TIS was somewhat responsive to single-dose levodopa treatment. Since TIS is an upper limb high-frequency-based measure, it cannot be detected during clinical assessment.

## 1. Introduction

Parkinson’s disease (PD) is a progressive, neurodegenerative disorder that is caused by the degeneration of dopaminergic neurons in the substantia nigra and characterized by motor symptoms. Common treatments for PD are levodopa and dopamine agonist tablets, deep brain stimulation (DBS), and levodopa/carbidopa intestinal gel (LCIG) [1]. Over the course of the disease, levodopa dose and timing of intake have to be adjusted to optimize the therapeutic effect [2]. DBS therapy’s effectiveness is enhanced by the selection of stimulation parameters [3].

Motor symptoms of PD are typically assessed using clinical scales such as the unified PD rating scale (UPDRS), which is used to evaluate the presence and severity of PD symptoms as well as symptom fluctuations. However, clinical scales-based measurements are not able to capture variations in symptoms continuously and they are insensitive to subtle changes. To reveal the full extent of patients’ condition and prevent recall and reporting bias, the symptoms need to be captured frequently, as well as both before and after medication [4].

The regularity of patterns can be measured by regularity statistics which are centered on various entropy measures [5]. The entropy measures, in a different context, have been an essential component of the quantitative development of thermodynamics, statistical mechanics, and information theory. In probability theory, entropy is defined [6] with regard to the amount of uncertainty and corresponds with intuition, in that systems with more random probability distributions have greater entropy. Entropy has also been considered as a critical “summary” statistic in nonlinear dynamical system analysis and chaos [7]. In statistics, Approximate Entropy (ApEn) is a technique to quantify the regularity, unpredictability, uncertainty of fluctuations over time-series data. A formula, based on Kolmogorov and Sinai (K-S) [8], to measure the entropy of a time series has been developed [9]; this formula has become the “standard” entropy and regularity measure for use with time-series data. However, since for a stochastic process K-S entropy is often infinite while ApEn is finite, ApEn can provide useful system information to distinguish differing stochastic processes.

There are different studies with the focus of quantifying PD symptoms’ regularity, variability, complexity, and the unpredictability of various signals using ApEn. In a study that measured regularity, it was found that the ankle joint movement signal of PD patients, when walking on a treadmill after levodopa medication, was more regular than before medication [10]. The regularity measure of the tremor signal was greater in groups of PD patients compared to healthy controls [11]. In the same way, the wrist movement signal in PD patients had higher regularity than healthy controls [12]. Research studies with alternate tapping and spiral performances [13,14] used ApEn as one of the calculated features to automate the process of visual interpretation of movement anomalies. In one study, the ApEn-based variability score was higher for the power of the speed signal in the dynamic PD group, compared to static PD group, while they performed a biking exercise [15].

In some studies, the timing variability was increased among patients when compared to healthy subjects. For instance, it was shown [16] that medicated PD patients had poorer timing control than those who were withdrawn from medication when modulating timing to an external stimulus. It was found [17] that measures of motor timing accuracy can discriminate PD patients from healthy controls.

In the context of spiral drawing analysis for PD with tablets, one recent study calculated a composite score by averaging speed and pressure, which was used to distinguish the different stages of PD [18]. Another study calculated a non-entropy-based spatial irregularity score as one of the indices to detect early PD [19], while another investigated the same indices for the adaptation to an iPad tablet [20]. To our knowledge, there has not been a particular investigation of ApEn for PD where subjects performed spiral tests, except in our last study [21]. The novelty of this study is that it investigates the clinical properties of the irregularity measure in the spiral drawings of PD patients and healthy controls, when the spiral drawing tests were performed during a single-dose levodopa study.

Our previous study [21] (denoted as ST1 in this paper) developed and evaluated the clinimetric properties of an ApEn score for measuring a PD-related temporal irregularity score (TIS) using digital spiral analysis. The upper limb temporal irregularities of 98 PD patients in different stages of PD and 10 healthy subjects in an open longitudinal 36-month study were evaluated. The test-retest reliability of the TIS was assessed. The long-term trend of the TIS after changing the treatment to LCIG pump was researched, and the responsiveness of the TIS before and after LCIG pump treatment was investigated. The results were compared to results from two other methods [22,23], which were related to the spatial components of spiral drawing and used to measure overall drawing impairments. However, the correlations between TIS and clinical ratings, the sensitivity of TIS to a single dose of levodopa over a course of test trials, and the reproducibility of the previously reported TIS properties have not been studied.

The purpose of the present study is to verify the results from ST1 and further investigate the clinimetric properties of TIS. The investigation was conducted using a new dataset, new patient groups, as well as another screen resolution, and during shorter term measurements.

This paper reports the properties of the temporal irregularity, including the differences in TIS between patients in different stages of PD and healthy subjects, and the test-retest reliability of TIS. In addition, the responsiveness of TIS to levodopa treatment during one-day, single-dose experiments and the correlations between TIS and clinical ratings are reported.

## 2. Materials and Methods

### 2.1. Participants

Nineteen PD patients and 22 healthy controls were recruited in a single center, open label, single-dose clinical trial in Uppsala, Sweden (Table 1) [24]. Written informed consent was given after approval by the regional ethical review board (in Uppsala, Sweden, reference number: 2015/100). No participant in the study had cognitive or visual problems to the extent to which it could influence their test performance.

### 2.2. Data Collection

The trial included a single levodopa-carbidopa dose experiment for the PD patients, where they were asked to perform a fine motor test (spiral drawing) using a smartphone before the dose, and at specific time intervals after the dose. Healthy controls were asked to perform the same test using the smartphone at specific time intervals. For the patients, the administered dose was 150% of their individual levodopa equivalent morning dose to follow transitions between Off, On, On with dyskinesia, and back to Off motor states. Up to 15 samples per PD patient were collected, one measurement at baseline (20 min prior to dosing), one at the time of dose administration (at time 0) and follow-up measurements at 15, 30, 45, 60, 80, 100, 120, 150, 180, 210, 240, 300, and 360 min after dose administration. The healthy controls were asked to perform the tests, up to eight times each, at time point 0 (first test) and then at 20, 40, 60, 80, 110, 140, and 170 min, without receiving any medication.

**Figure 1:**
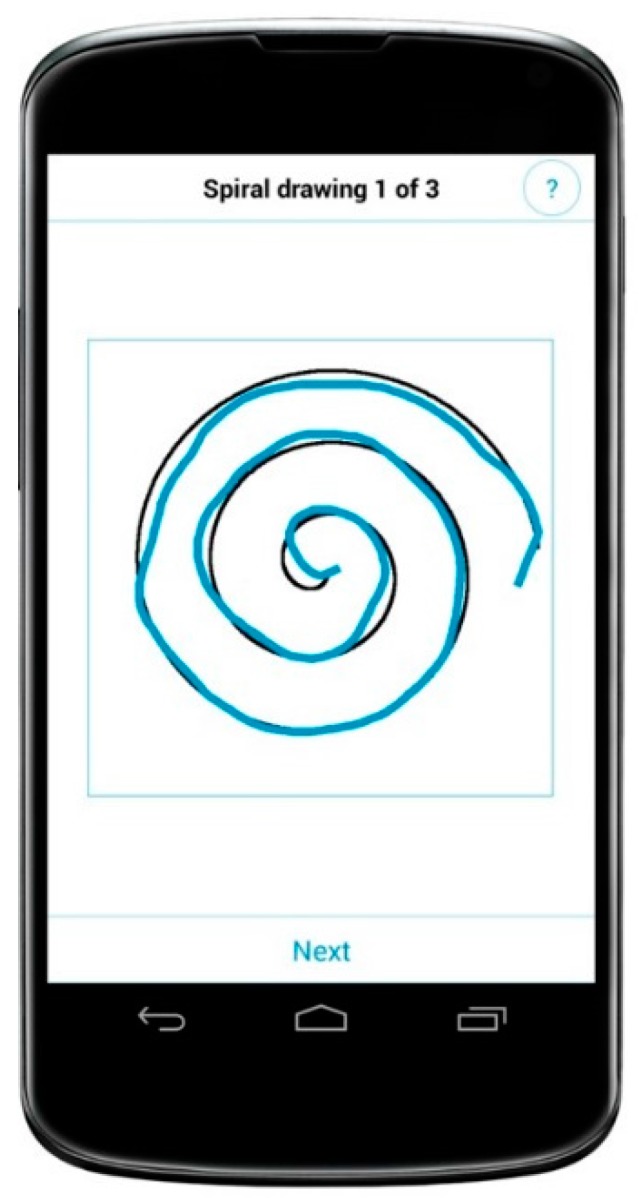
Implementation of a fine motor test (spiral drawing) on the smartphone.

On each test occasion, subjects performed spiral drawings tests using a smartphone (Figure 1). The smartphone had a 4” (86 mm × 53 mm) touch screen with a screen resolution of 480 × 800 pixels (~233 PPI pixel density) and recorded both positions (*x* and *y* coordinates) and time-stamps (in milliseconds) of the pen tip. The device sampling was event-based, which means a sensor event was generated every time the sensor values *x* and *y* were changed. The subjects were instructed to be seated on a chair and perform the tests using an ergonomic pen stylus with the device placed on a table and supporting neither hand nor arm. During the spiral tests, the subjects were instructed to trace a pre-drawn Archimedes spiral as fast (within 10 s) and accurately as possible, from the center out, using the dominant hand. The drawing was repeated three times per test occasion. The total number of occasions with the smartphone for PD patients was 240, and for healthy controls it was 176.

Along with smartphone-based measurements, patients were video recorded while performing standardized motor tasks according to UPDRS at corresponding time points.

### 2.3. Clinical Assessments of Motor Symptoms

The recorded videos were presented in a randomized order to three movement disorder specialists so that the ratings were blinded with respect to time from dose administration. The specialists rated six UPDRS-part III (motor examination) items including UPDRS #23 (finger tapping), UPDRS #25 (rapid alternating movements of hands), UPDRS #26 (leg agility), UPDRS #27 (arising from chair) and, UPDRS #29 (gait), UPDRS #31 (bradykinesia), according to the definitions of the motor examination part of the UPDRS [25]. The specialists also rated dyskinesia on a severity scale from 0 to 4 [26]. For every scale, mean scores per time point for the three specialists were calculated and used in subsequent analysis.

### 2.4. Feature Extraction

Since the sampling rate was event-based and at a higher screen resolution compared to the sampling rate obtained in ST1, the data from the new study were down-sampled by extracting every second data point, in order to yield an effective average sampling rate comparable to that in ST1. The digitized spiral signals were processed using ApEn to generate a TIS. Initially, drawing speed was calculated as a rate of spatial change with respect to time, using the following formula:

(1) $$DS=\frac{\sqrt{{\left(x_{i+1}-x_{i}\right)}^{2}+{\left(y_{i+1}-y_{i}\right)}^{2}}}{t_{i+1}-t_{i}}$$

where *x* is the horizontal coordinate of pixels on the screen, *y* is the vertical coordinate, and *t* is the time in seconds. The ApEn technique was then applied to drawing speed (*DS*) signals to generate the TIS.

Theoretically, ApEn is a statistical method that measures the complexity and repeatability of patterns within a signal [27]. Signals which contain a single frequency component are associated with relatively small ApEn values, whereas more complex signals which contain multiple frequency components are associated with high ApEn values, indicating a high level of irregularity. ApEn reflects the similarity between a chosen window of a given duration and the next set of windows of the same duration. The ApEn formula is given by [28]:

(2) $$ApEn\left(m,r,N\right)=\frac{1}{N-m+1}\overset{N-m+1}{\underset{i=1}{\sum }}\log C_{i}^{m}\left(r\right)-\frac{1}{N-m}\;\overset{N-m}{\underset{i=1}{\sum }}\log C_{i}^{m+1}\left(r\right)$$

where *m* is an integer that specifies the length of the window being compared, *r* is a positive real number that specifies a filtering level, *N* is the total number of data samples, and *C*(*r*) is the correlation integral. *m* and *r* must remain fixed during all calculations. Searching for the best *m* and *r* configurations across a set of commonly used values (*m* = 2, 4 and *r* = 0.1, 0.2), the ApEn value was computed for all possible resulting combinations. The criteria to select best *m* and *r* were based on the results for higher test-retest reliability, higher sensitivity, higher correlations between TIS and clinical ratings, and better separation of the patients’ groups from healthy subjects. After experimentation, *m* was set to 2 and *r* was set to 0.2 (20% of the standard deviation of the signal). To increase the stability of the TIS, the extracted ApEn value was then divided by the total drawing completion time. Since there were three trials that were performed during each spiral test, the average of the three TIS values was calculated and used in the following analysis.

### 2.5. Statistical Analysis

To be able to conduct a comparative analysis with groups of patients in ST1, three groups of patients were defined based on the Hoehn and Yahr scale [29] and presented as a median ± interquartile range with the following criteria: Early (≤2 ± 1), Intermediate (3 ± 0), and Advanced (≥4 ± 1). Some patients in Hoehn and Yahr stage 3 were separated, such that patients with less than 5 years’ levodopa treatment were included in the early group and those with more than 5 years’ treatment were included in the intermediate group.

The significance of the mean TIS, as well as the age across the five subject groups of healthy, early, intermediate, advanced, and all patients was investigated using Linear Mixed Effect (LME) models based on a restricted maximum likelihood estimation method. The difference in gender between healthy subjects and patients was investigated with the proportion test using Fisher’s exact method. To evaluate the consistency of the TIS, the test-retest reliability of the TIS was assessed using the intra-class correlation coefficient (ICC) between the three spiral tests. The ability to detect the change from baseline (before medication) to follow-up time points when patients were on medication was assessed by effect sizes. To calculate effect sizes, ANOVA models were fitted for each time point after the baseline test; first test and second test; first test and third test, and so on. A high effect size indicates that a scale is sensitive in detecting the treatment response [30]. To investigate the relation between TIS and specialists’ visual assessments of PD symptoms, the correlations between TIS and clinical rating scores were assessed by Pearson correlation coefficients.

## 3. Results

**Figure 2:**
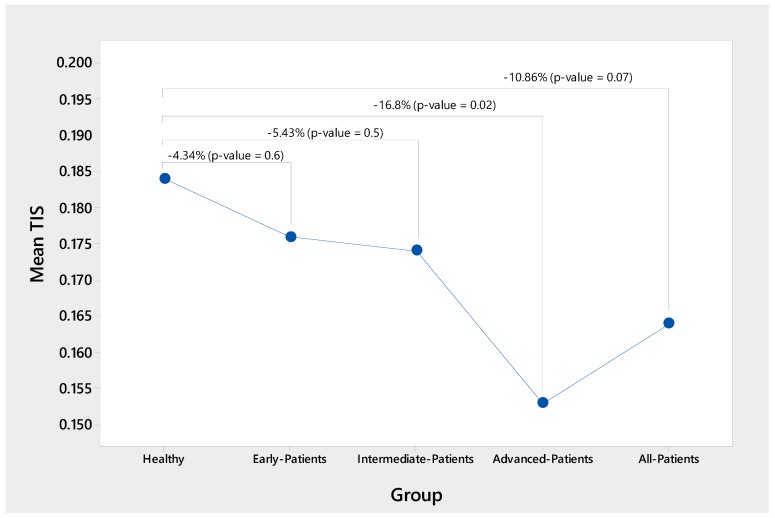
Linear mixed effect (LME) fixed effects coefficients of the Temporal Irregularity Score (TIS) (with age as covariate) across the five subjects groups. *p*-values (groups: Early, Intermediate, Advanced, and All patients) with respect to healthy subjects were: 0.6, 0.5, 0.02, 0.07. Number of participants: Healthy (*n* = 22), Early (*n* = 7), Intermediate (*n* = 8), Advanced (*n* = 4), All patients (*n* = 19). Number of observations: Healthy (*n* = 176), Early (*n* = 93), Intermediate (*n* = 90), Advanced (*n* = 57), All patients (*n* = 240).

When assessing the differences of TIS between tests performed by patients at different PD stages and healthy controls, the values were found to be significantly different between healthy subjects and advanced patients (*p*-value = 0.02), similar to the results in ST1 (*p*-value < 0.05). They were not significantly different between the healthy subjects and the whole patient group (*p*-value = 0.07). There was a significant difference (*p*-value = 0.00) in age between the healthy controls (mean = 64 years) and the whole patient group (mean = 71 years). The results for differences of mean TIS when considering the patient groups as main effect and the age as a covariate are shown in Figure 2.

There was no significant difference (*p*-value = 0.52) in gender distribution between healthy controls and patients. The test-retest reliability of TIS was 0.81 (ICC), which was similar to the results in ST1 (*R* = 0.74) and indicated the consistency of TIS between the three spirals.

**Figure 3:**
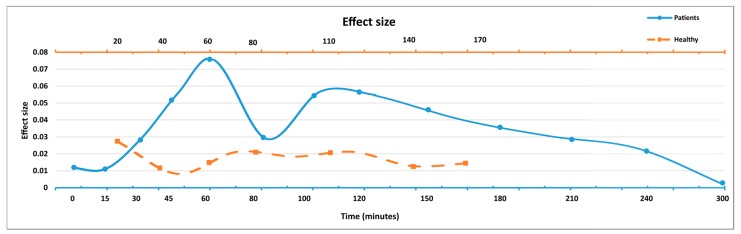
Sensitivity assessment of TIS across the test occasions. The lower *x* axis represents the minutes after taking the levodopa dose for patients and the upper *x* axis represents the tests time points for healthy subjects. The first data point in the *x* axis represents the difference in scores between first (baseline) and second measurements; the second data point represents the difference in scores between first and third measurements, and so on. Number of patients for periods: 0 (*n* = 19), 15 (*n* = 19), 30 (*n* = 19), 45 (*n* = 19), 60 (*n* = 18), 80 (*n* = 18), 100 (*n* = 18), 120 (*n* = 18), 150 (*n* = 18), 180 (*n* = 17), 210 (*n* = 15), 240 (*n* = 13), 300 (*n* = 9). Test 15 at 360 min contained only one patient, which was not enough to be included in this analysis. Number of healthy subjects for periods: 20 (*n* = 22), 40 (*n* = 22), 60 (*n* = 22), 80 (*n* = 22), 110 (*n* = 22), 140 (*n* = 22), 170 (*n* = 22).

When assessing the sensitivity of TIS to the treatment response for patients, the results indicated that the patients’ scores can capture some effects of the medication. Effect sizes (Figure 3) were increased from 15 min after dose intake to 60 min, indicating the changes in symptoms from off to On/dyskinesia. Likewise, from 100 min to 300 min the effect sizes were decreased, indicating wearing off effects. At 80 min, the effect size was smaller than could be expected from the medication trend.

For healthy subjects, the effect sizes were smaller than the effect sizes of patients. At the first tests, there were some variations in the effect sizes but they tended to converge around a fixed point during later tests, which could be an indication of the learning effect after repeating the spiral drawings.

The correlations between TIS and mean clinical rating scores (UPDRS, Dys) were weak: −0.18 for UPDRS item 23 (finger tapping), −0.11 UPDRS item 25 (rapid alternating movements of hands), −0.29 for UPDRS item 26 (leg agility), −0.24 UPDRS item 27 (arising from chair), −0.20 for UPDRS item 29 (gait), −0.10 for UPDRS item 31 (bradykinesia), and −0.31 for Dys (Dyskinesia).

## 4. Discussion

In this study, we verified some clinimetric properties of TIS which were proposed and evaluated in a previous study [21]. As in the previous study, the extracted TIS in the present study was significantly different between the spiral drawings drawn by patients in early, intermediate, and advanced stages, as well as between those drawn by patients and healthy subjects. The test-retest reliability of TIS was good. Referring to the results in the previous study, TIS increased with worsening disease severity. This can also be observed in the results from the present study, where the TIS mean value was significantly smaller in advanced patients when compared to healthy subjects (Figure 2). In this study, the sensitivity of TIS to the treatment response during single-dose experiments from before treatment to 300 min after levodopa intake was calculated. Previously [21], it was calculated for week-long periods with four spiral drawing occasions per day before switching to LCIG treatment, as well as after treatment. TIS had a greater effect size (0.078) compared to the effect sizes of the other two methods [22,23]. In the current study, the TIS effect sizes (Figure 3) were calculated for test periods up to 300 min after the levodopa dose for patients and up to 170 min of test trials for healthy subjects. The results in the present study show some but weak sensitivity to single-dose levodopa, whereas the sensitivity after switching to LCIG in the previous study was strong.

In the previous study, clinical evaluation was not possible since the limitations were related to the data collection scheme, where subjects repeatedly used the touch screen device in their home environments without clinical supervision and therefore there were no corresponding clinical ratings. However, in the current study, the collected data were timestamped and the videos of the patients were rated by movement disorder specialists, which made it feasible to investigate the possible correlations between TIS and clinical scores. We found that correlations between TIS and clinical rating scores in this study were weak. A reason for this could be that TIS was related to rapid temporal fluctuations during spiral drawing, since the ApEn technique provides a high resolution signal by partitioning it into a smaller set of windows and sliding the windows throughout the signal. This enables the TIS to measure high-frequency irregularities in spiral drawing speed in the order of milliseconds, where visual assessment of these irregularities can not be detected.

## 5. Limitations

A limitation of the study was that, after grouping the patients based on the disease severity, not many patients remained in each group compared to the number of healthy subjects. The results of this study suggest applying the current methodology to a larger dataset including more subjects at various stages of medication, and subjects at various years of treatment. The age distribution was different, as healthy controls were younger than patients. The sensitivity results in this study were much higher for clinical rating scores than for TIS. Sensitivity to LCIG in our previous study was strong, while sensitivity to single-dose levodopa was weak. This indicates that TIS might be more useful in long-term diagnostic tools rather than in detecting the single-dose levodopa response. TIS was weakly correlated to clinical symptom ratings, and may contribute together with other measures in combined scores.

## Figures and Tables

**Table 1:** Characteristics, mean (standard deviation) of patients and healthy subjects. UPDRS IV is the complications of therapy questions answered by patients regarding dyskinesias, clinical fluctuations and other complications.

	Gender	Mean Age (years)	Hoehn and Yahr Stage	Years with the Disease	Years on Levodopa	Levodopa Equivalent Dose	UPDRS IV
**Patients**	14 males 5 females	71.4 (6.3)	3.1 (0.8)	10 (6.8)	10 (6.8)	183.3 (55.9)	6.2 (3.1)
**Healthy controls**	16 males 6 females	64.2 (7.4)	NA	NA	NA	NA	NA
***p*-value**	0.62	0.00	NA	NA	NA	NA	NA
